# Stroke risk factors, subtypes and outcome in elderly Thai patients

**DOI:** 10.1186/s12883-021-02353-y

**Published:** 2021-08-20

**Authors:** Chatpol Samuthpongtorn, Tul Jereerat, Nijasri C. Suwanwela

**Affiliations:** 1 Faculty of Medicine, Chulalongkorn University, Bangkok, Thailand; 2 Division of Neurology, Department of Medicine, Chulalongkorn Comprehensive Stroke Center, King Chulalongkorn Memorial Hospital, Bangkok, Thailand

**Keywords:** Stroke, Outcomes, Risk factors, Stroke classification, Elderly, Thailand

## Abstract

**Background:**

Nowadays, the number of elderly has steadily increased annually.

Elderly patients with ischemic stroke often have worse outcomes than younger patients. However, there has not been a study of ischemic stroke in the elderly in Thailand. A better knowledge of the risk factors, subtypes, and outcomes of strokes in the elderly may have significant practical implications for the aged society in the future. The objective of the study was to assess the risk factor, stroke subtypes, and outcome of stroke in the elderly compared to the younger patients.

**Method:**

All patients presented with acute ischemic stroke and transient ischemic attack (TIA) aged over 45 years who were admitted in the Stroke unit between November 1st, 2016 and December 31st, 2017 were retrospectively studied.

**Result:**

Five hundred forty-two patients were included. The average age was 68.78 ± 12.03, 44.8% of them were male. 186 (34.3%) patients were 75 or older. Cardioembolism was found to be the most common cause of ischemic stroke in 156 patients (28.8%) and was associated with poor outcome. Patients who were 75 or older had significantly worse outcomes in all categories including NIHSS at discharge, modified Rankin scale, length of stay and the number of deaths compared to the younger group. Atrial fibrillation was the risk factors associated with older age with OR 3.861 (*p* value< 0.001). Aged 75 years or older, atrial fibrillation, more NIHSS score on admission and history of the previous stroke were the risk factors associated with a patient’s death.

**Conclusion:**

The elderly who are 75 years or older accounts for more than one-third of ischemic stroke in our study. Stroke in the elderly correlates with higher mortality and poorer outcome. Cardioembolism related to atrial fibrillation is the major cause of stroke in this population.

## Introduction

Stroke is one of the leading causes of death and disability in Thailand. The incidence of strokes in Thailand has been increasing annually. The number of new stroke cases are approximately 250,000 cases and accounts for more than 50,000 deaths each year. The prevalence of stroke is estimated to be 1.88% among adults 45 years and older [1]. It carries the highest rank for disease burden in Thailand measured by disability-adjusted life years (DALYs) lost in females and the third in males [2].

Nowadays, an aging phenomenon is one of the most important global challenges. The number of the elderly has steadily increased and it is expected that Thailand will be faced with an aged society of which more than 20% of the population will be older than 60 in 2021 [3, 4].

Major risk factors of stroke include age, ethnicity, hypertension, diabetes mellitus, smoking, metabolic syndrome and atrial fibrillation. There are many subtypes of stroke and some subtypes are highly related to age, for example, atherosclerosis, atrial fibrillation and small vessel disease [5–7]. Moreover, elderly patients with ischemic stroke often have worse outcomes than younger patients [8, 9]. Ischemic stroke has not been investigated in the elderly in Thailand. Hence, a better knowledge of the risk factors, subtypes and outcome of strokes in the elderly may have significant practical implications for early detection, stroke prevention and clinical management for the aged society in the future. The objective of the study was to assess the risk factors, stroke subtypes and outcome of stroke in the elderly compared to the younger patients.

## Method

All patients presented with acute ischemic stroke and transient ischemic attack (TIA), aged over 45 years who were admitted to the Stroke unit, King Chulalongkorn Memorial Hospital between November 1st, 2016, and December 31st, 2017, were retrospectively reviewed. Patients with hemorrhagic stroke and cerebral venous thrombosis were excluded. Ischemic stroke was diagnosed by clinical history, physical examination and confirmed by neuroimaging. TIA was defined as any focal cerebral ischemic event with symptoms lasting less than 24 h. Electronic medical records and neuroimaging studies were reviewed. Clinical parameters considered were age, sex, vascular risk factors, associated comorbidity, stroke etiology and functional outcome. Data on TOAST classification, NIHSS score and Modified-Rankin scale were also collected. All of the data were evaluated by neurologists at the Stroke Unit. The data that we have collected are the data at the hospital on the day the patient was discharged. The study collected outcomes at discharge date which included NIHSS score, Modified-Rankin scale, length of stay and the number of deaths. These variables were defined as short-term outcomes. Based on the TOAST criteria, ischemic stroke was classified into 5 categories: large-artery atherosclerosis, cardioembolism, lacunar infarction, other determined and undetermined etiologies. The study was approved by the Institutional Review Board of the Faculty of Medicine, Chulalongkorn University (IRB 037/63).

### Ethics

This study met the criteria of Expedited review from International Ethical Guidelines for health-related research Involving Human 2016 [10]. Two medical students (CS and TJ) collected the medical records of the patients between November 1, 2016, and December 31, 2017. Information were summarized by the neurologists. These chart reviews gathered information on a specific medical condition and set of patient characteristics. The data was kept anonymous without name or contact of the participant.

### Statistical analysis

Statistical Package for the Social Science for Windows Version 21(SPSS 21) was used for all analyses. Categorical data were presented as number and percentage. Continuous variables were expressed as mean +/− standard deviation. Categorical data were analyzed by the chi-square test. The Mann-Whitney U test was used to compare the continuous variables. Multivariate logistic regression analysis was performed between independent variables that were considered to be associated with stroke subtypes. *P* value less or equal to 0.05 was considered to be statistically significant.

## Results

There were 542 patients in the study. Fifty-four (9.96%) presented with TIA, and 488 (90.04%) had ischemic stroke. The average age of the patients was 68.78 ± 12.03 of which 44.8% were male. There were 186 (34.3%) patients who were 75 or older. The most common risk factor was hypertension, followed by dyslipidemia and diabetes mellitus. Baseline characteristics of the patients are shown in Table 1.

**Table 1:** Characteristics of patients stratified by age

Characteristic	Patients aged 45–74 years	Patients aged ≥ 75 years	Total	*p*-value
	*n* = 356	*n* = 186	*n* = 542	
Male: n (%)	144 (40.4%)	99 (53.2%)	243 (44.8%)	0.005
TIA: n (%)	34 (63%)	20 (37%)	54 (9.96%)	0.657
Risk factors: n (%)				
History of diabetes mellitus	124 (34.8%)	76 (40.9%)	200 (36.9%)	0.167
History of hypertension	248 (69.7%)	150 (80.6%)	398 (73.4%)	0.006
History of dyslipidemia	203 (57.0%)	113 (60.8%)	316 (58.3%)	0.403
Atrial fibrillation	51 (14.3%)	72 (38.7%)	123 (22.7%)	< 0.001
Smoking	27 (7.6%)	5 (2.7%)	32 (5.9%)	0.022
Previous stroke	63 (17.7%)	50 (26.9%)	113 (20.8%)	0.012
Previous TIA	11 (3.1%)	3 (1.6%)	14 (2.6%)	0.301
Previous MI	31 (8.7%)	23 (12.4%)	54 (10.0%)	0.177
NIHSS on admission (mean ± SD)	6.36 ± 0.33	7.20 ± 0.57	6.65 ± 0.29	0.745
Stroke subtype: n (%)				
Large artery atherosclerosis	100 (28.1%)	30 (16.1%)	130 (24.0%)	0.005
Cardioembolism	75 (21.1%)	81 (43.5%)	156 (28.8%)	< 0.001
Small vessel occlusion	108 (30.3%)	41 (22.0%)	149 (27.5%)	0.085
Other determined	7 (2.0%)	1 (0.5%)	8 (1.5%)	0.498
Undetermined	66 (18.5%)	33 (17.5%)	99 (18.3%)	0.859

**Fig. 1:**
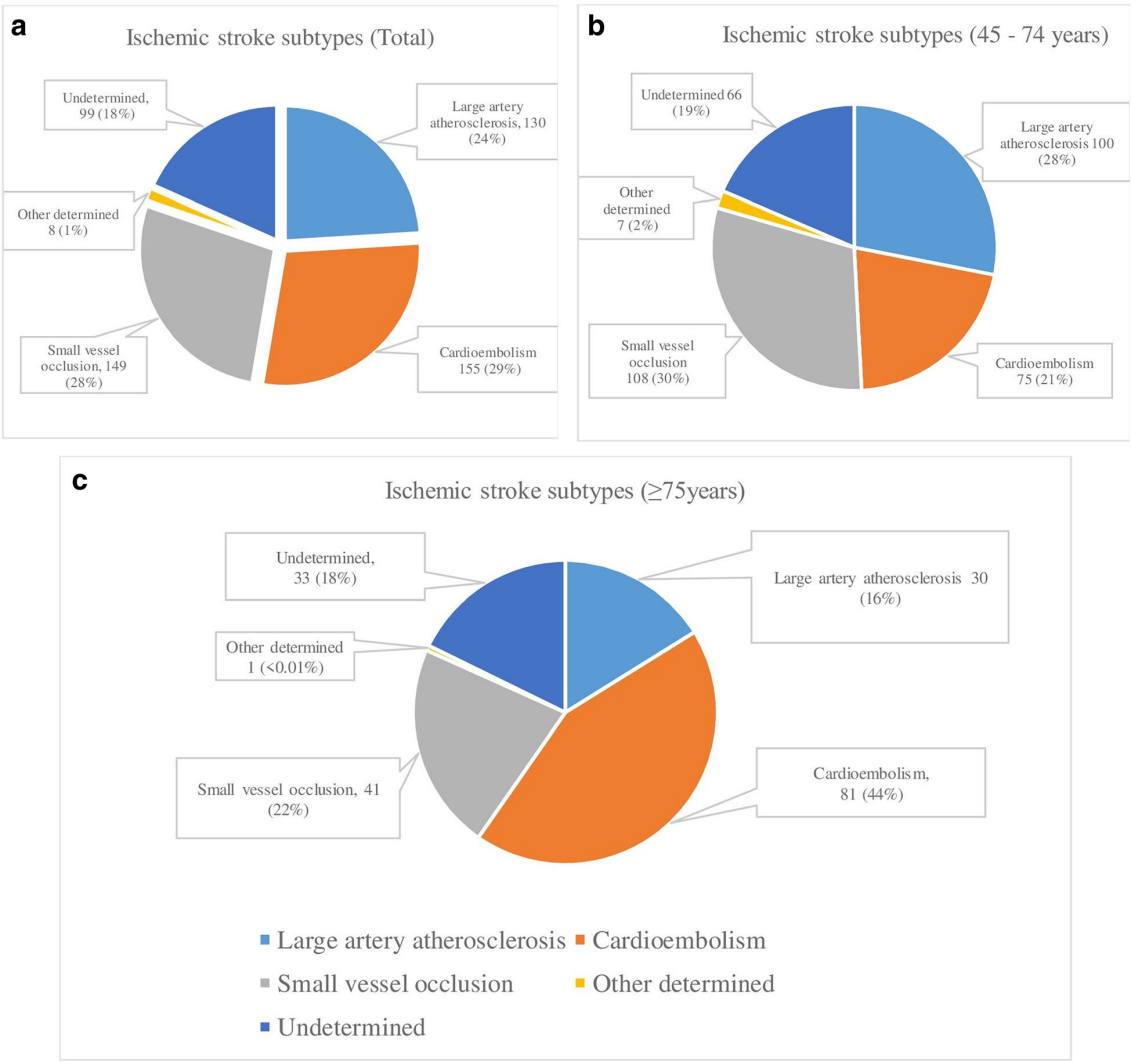
Prevalence of stroke subtypes **A** in the study **B** in patients aged 45–74 years old **C** in patients aged 75 years or older

According to the TOAST classification, cardioembolism was found to be the most common cause of ischemic stroke in 156 patients (28.8%), followed by small vessel disease in 149 patients (27.5%), and large vessel atherosclerosis in 130 (24.0%). Prevalence of stroke subtypes was shown in Fig. 1.

**Fig. 2:**
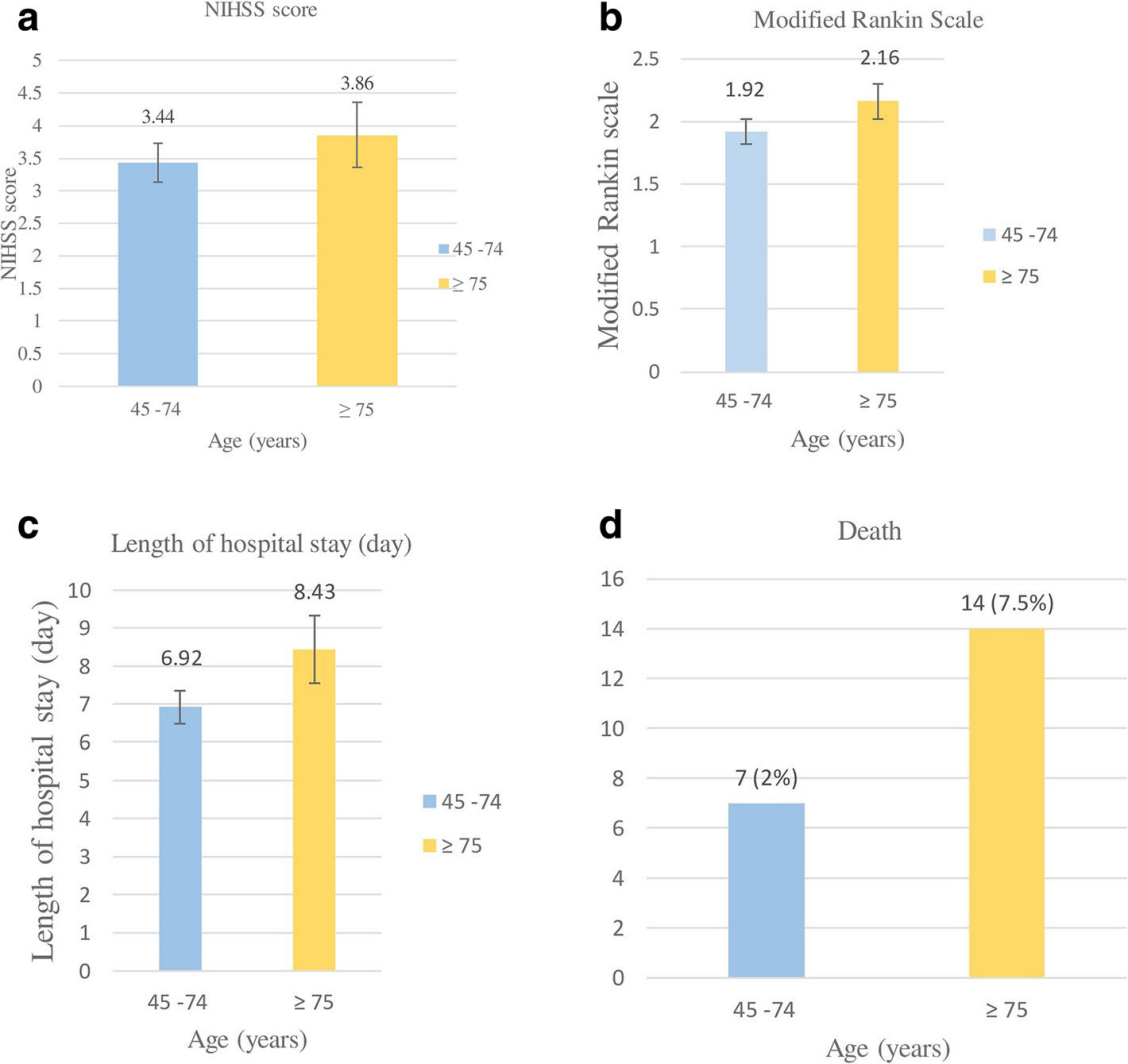
Outcome of patients at discharge **A** NIHSS score **B** Modified Rankin Scale **C** Length of hospital stay and **D** Number of death

There were significantly higher percentages of hypertension and atrial fibrillation among patients who were 75 or older. Eighty one patients (43.5%) had cardioembolism which was the most common subtype of ischemic stroke followed by small vessel disease in 41 patients (22.0%). Stroke outcomes were described by NIHSS at discharge, modified Rankin scale, length of hospital stay and the number of deaths. Patients who were 75 and older had significantly worse outcomes for all categories compared to the younger age group Outcome of the patients at discharge was shown in Fig. 2.

In the multivariate analysis, the stroke risk factor associated with patients who were 75 and older was atrial fibrillation. The OR for atrial fibrillation was 3.861 (*p* value< 0.001) (Table 2).

**Table 2:** Multivariate analysis for risk factors associated with patients aged more than 75 years old

	Patients aged ≥ 75 years	
	*P* values	Odds ratio(95% CI)
sex	0.046	1.478 (1.007–2.168)
History of diabetes mellitus	0.659	1.097 (0.727–1.657)
History of hypertension	0.117	1.485 (0.905–2.436)
History of dyslipidemia	0.852	1.041 (0.684–1.584)
Atrial fibrillation	< 0.001	3.466 (2.256–5.324)
Smoking	0.242	0.545 (0.198–1.506)
Previous stroke	0.09	1.481 (0.940–2.334)
Previous TIA	0.777	0.826 (0.221–3.091)
Previous MI	0.335	1.350 (0.733–2.487)

Twenty-one patients died during hospitalization. Among these, 14 patients (66.7%) were 75 years and older. Risk factors that were associated with death were aged 75 years and older, atrial fibrillation, high NIHSS score upon hospital admission, and history of previous stroke (Table 3).

**Table 3:** Factors associated with death during hospitalization

Factor	Survive	Death	*p*-value
	*n* = 521	*n* = 21	
Age ≥ 75: n (%)	172 (33.0%)	14 (66.7%)	0.001
Male: n (%)	231 (44.3%)	12 (57.1%)	0.247
Risk factor: n (%)			
History of diabetes mellitus	193 (37.0%)	7 (33.3%)	0.73
History of hypertension	381 (73.1%)	17 (81.0%)	0.426
History of dyslipidemia	307 (58.9%)	9 (42.9%)	0.143
Atrial fibrillation	111 (21.3%)	12 (57.1%)	< 0.001
Smoking	32 (6.1%)	0 (0.0%)	0.242
Previous stroke	105 (20.2%)	8 (38.1%)	0.047
Previous TIA	13 (2.5%)	1 (4.8%)	0.522
Previous MI	50 (9.6%)	4 (19.0%)	0.156
NIHSS on admission (mean ± SD)	6.301± 0.284	14.789± 1.770	< 0.001
Stroke subtype: n (%)			
Large artery atherosclerosis	127 (24.4%)	3 (14.3%)	0.602
Cardioembolism	144 (27.6%)	12 (57.1%)	0.002
Small vessel occlusion	148 (28.4%)	1 (4.8%)	0.021
Other determined	7 (1.3%)	1 (4.8%)	0.652
Undetermined	95 (18.2%)	4 (19.0%)	0.788

## Discussion

We retrospectively studied the risk factors, subtypes, and outcome of ischemic stroke in a single tertiary care center in Thailand. The mean age of our patients was 68.8 years, and 34.3% were 75 years and older. The proportion of elderly in this study is consistent with the data of general Thai population where 38.7% were elderly [11]. According to the national stroke data, 31.0% of stroke patients were over 75 years old [12]. The prevalence of elderly stroke patients varied among countries and studied population. A study of France found that 28% of stroke patients were elderly [13] and the absolute number of first-ever strokes increased by 47% in the patients over 75 years old [13]. Another hospital-based prospective study conducted in China found that 22% of the patients were over 75 years old [14] whereas a multicenter study conducted in Mexico showed that approximately 40% of stroke patients were older than 75 years [15]. The mortality rate among elderly patients in our study was 3.8% which we followed up patient at the hospital discharge day. In contrast, there is the higher mortality rate in previous studies due to longer period of time of following up patients [13].

Among the elderly stroke patients, hypertension and atrial fibrillation were the 2 most prominent risk factors. Hypertension was the most prevalent risk factor and documented in 73.4% of total patients in the entire cohort. This risk factor was also noted to be prevalent in many Asian studies including studies from northern China and Southeast Asia [16, 17]. Our study found that 38.7% of the elderly patients had atrial fibrillation compared to only 14.3% in younger patients. This correlated well with the stroke subtype of which the majority of the elderly patients had cardioembolism. Atrial fibrillation is strongly associated with increased age which was similar to findings reported by other studies [11, 18, 19].

The most common subtype of ischemic stroke among the elderly in this study was cardioembolism which accounted for more than 40% of the cases. Atrial fibrillation was responsible for most of them. In contrast, cardioembolic stroke was responsible for only 20% of those who were younger than 75 years old. This high prevalence of atrial fibrillation may be partly related to the high rate of EKG monitoring among our stroke patients in the stroke unit. EKG monitoring is routinely performed for at least 24 h in all stroke cases in our institution. Moreover, in those who had clinical suspicion of cardioembolic stroke, Holter monitoring was recommended for further evaluation. Alternatively, large artery atherosclerosis was relatively more common in the younger patients. In both age groups, the proportion of small vessel occlusion was not significantly different.

Regarding the outcomes, patients aged 75 years and older had 2.5 times higher mortality rate and poorer outcome than patients aged below 75 years which is consistent with previous studies [20]. The discharged NIHSS scores, mRS, and length of hospital stay were also significantly higher among the elderly. These unfavorable outcomes may be related to a greater severity of stroke among the elderly as measured by the initial NIHSS scores. Moreover, the elderly patients tended to have a higher prevalence of risk factors as well as other comorbidities such as atrial fibrillation and previous stroke. We also observed that cardioembolism was the major cause of stroke in the elderly and this stroke subtype was associated with a poor outcome. Previous studies also demonstrated that patients with cardioembolic stroke in the atrial fibrillation group had a higher mortality rate and worse outcome when compared to non-atrial fibrillation group [14, 21]. Because of the higher prevalence of atrial fibrillation in the elderly patients [11, 18, 19], they tended to have a higher mortality rate and worse outcome.

Our study highlighted the importance of stroke among the elderly as we are entering ageing society [3, 4]. In this study, we used the age of 75 years as the cut-off point which is in line with other recent studies that focused on the treatment of the very old population [11, 13]. Hypertension was the most common risk factor and cardioembolic stroke was the most common stroke subtype in the elderly and was associated with poor outcome. Thus, it is very important to detect atrial fibrillation among the elderly in order to secondary prevent stroke and manage the patient appropriately.

This study has some limitations. Some confounding factors may not be fully evaluated due to the retrospective nature of the study. For example, the medical records were not designed for the study. Some risk factors which there was no direct impact on patient care during admission (e.g. history of alcohol use, obesity) were not recorded. Also, the medical records were evaluated at the Stroke Unit by more than one neurologist. The data in each medical records were different. Second, although EKG monitoring was performed in all patients, further evaluation by Holter monitoring were only done in suspected patients. Therefore, it is possible that the number of patients who had atrial fibrillation may have been underestimated. Finally, the outcomes that have been collected were at the day of hospital discharge which is the short-term outcomes. The long-term outcomes are not reported in this study.

## Conclusion

Elderly who were 75 years and older accounted for more than one-third of ischemic stroke patients in our study. Stroke in the elderly correlated with higher mortality and poorer outcome. Cardioembolism related to atrial fibrillation was the major cause of stroke in this population.

## Data Availability

Requests to access the datasets should be directed to [Chatpol Samuthpongtorn, jamie15006@gmail.com]”.

## Abbreviations

CT: Computed tomography
EKG: Electrocardiogram
mRS: Modified Rankin Scale
NIHSS: National Institute of Health Stroke Scale
SD: Standard deviation
TOAST: Trial of Org 10,172 in Acute Stroke Treatment
